# Psychologists’ reflections on a trans and gender-diverse group at Baragwanath Hospital

**DOI:** 10.4102/sajpsychiatry.v31i0.2327

**Published:** 2025-06-13

**Authors:** Coenderaad J.F. Jacobs, Najeebah Y. Noorbhai

**Affiliations:** 1 Department of Health, Chris Hani Baragwanath Academic Hospital, Johannesburg, South Africa; 2 Department of Psychiatry, Faculty of Medicine, University of the Witwatersrand, Johannesburg, South Africa

**Keywords:** group therapy, psychologist, psychotherapy, public health, reflective, trans and gender diverse

## Abstract

**Background:**

Statistics indicated that, in 2019, over 3 million of South Africa’s population of 58 million people presented as gender non-conforming. This is challenging, as in South Africa, the Western binary concept is still prevalent. In light of the above, South African research on trans and gender-diverse (TGD) interventions is essential. There is limited research on existing TGD group interventions in a South African public context.

**Aim:**

This article aimed to report on the personal reflections of the two clinical psychologists who co-facilitated a support group for TGD people at Chris Hani Baragwanath Academic Hospital (CHBAH). This study highlights the importance of group intervention in particular.

**Setting:**

Chris Hani Baragwanath Academic Hospital in South Africa.

**Methods:**

A reflexive thematic analysis was utilised. Thoughts and self-reflective themes discussed after each group were included. No participants or instruments were utilised in this research.

**Results:**

The following themes were identified in this research: gaps in our knowledge, the role of facilitators, misgendering and gender fluidity, collective self-esteem, corrective experience and basic rights.

**Conclusion:**

This group contributed to our learning and development as facilitators. The role of reparation and empathy as facilitators was highlighted.

**Contribution:**

The results support the importance of support groups for TGD individuals in public healthcare as well as the further development of healthcare professionals. Because of a scarcity of psychological services rendered in public hospitals to TGD people, this article may potentially be a rich source of data for future TGD support groups and/or services.

## Introduction

Trans and gender-diverse (TGD) refers to ‘an umbrella term for people whose gender identity is different form their assigned sex at birth and/or who express their gender in non-traditional ways’.^1^ It is unrelated to a person’s sexuality, preference and/or attraction.^1^ According to the Psychological Society of South Africa (PsySSA) guidelines, *transgender* refers to ‘people who challenge society’s view of gender as fixed, dichotomous and inextricably linked to one’s biological sex’ (PsySSA, p. 63).^2^ Gender-diverse refers to individuals who do not conform to societal or cultural expectations of men and women.^3^ The incongruence between gender expression and societal norms has resulted in events of discrimination, harassment, victimisation, feeling devalued and negative beliefs towards one’s own gender identity.^4^ Psychological Society of South Africa emphasises the importance of awareness of these needs and other challenges faced by sexual and gender-diverse individuals.^5^

Misgendering has negative effects on TGD individuals.^6^ These negative effects include a lowered self-esteem, feelings of discontent, distress, despair and feeling nullified.^7^ These feelings can be evoked in a therapeutic space when a therapist uses an incorrect pronoun and/or an individual’s birth name as opposed to their real name. The best practice is to refrain from this.^6^ Therapists have their own unconscious biases which stem from social stereotypes; therefore, there is a probability of unintentional misgendering to occur and is inevitable. However, this does provide an opportunity for rapport building, repair or reparative work^6^ by opening up dialogue between therapist and group members and between members.

Traditional binary gender norms can cause TGD individuals significant physical health problems^8^ and mental distress. Physical health outcomes like elevated blood pressure and heart rates have been linked to discrimination and characterised as a stress response.^8^ De Vries et al.^3^ added that these health challenges further originate from structural factors, biases in the healthcare system, community factors, interpersonal factors, as well as from the ignorance of health professionals.

Individuals who identify as TGD are often rejected by families and communities alike and are often marginalised because of their gender non-conforming behaviour.^9^ Gender identity and sexual orientation that deviates from cisnormative views is often a basis for discrimination.^9^

Mental health implications include depression, anxiety, trauma and stress-related symptoms and occasional psychotic symptoms.^8^ External events (such as discrimination), the anticipation and expectation of external events, as well as internalised transphobia have a negative impact on one’s ability to cope, which reduces one’s resilience.^10^ According to the minority stress model, these factors create cumulative psychological distress.^11^

According to Dikey and Loewy,^12^ support groups for TGD individuals offer a crucial service that may not be available through individual psychotherapy. Support groups for TGD individuals have been proven to decrease social isolation, as well as to alleviate psychosocial stressors experienced by this population.^13^ Mallory et al.^14^ emphasised the therapist’s role in encouraging a sense of control and liberty to explore and express gender roles. Support offered to group members may be from the facilitators or the other group members who provide support based on their own personal experiences.^15^ In addition to support, Mallory et al.^14^ stated that group psychotherapy also offers validation to individuals and normalises the process of transitioning.

Having an effective support system assists TGD individuals in rebuilding self-worth, which can potentially prevent the above-mentioned symptoms from developing.^8^ In addition, a person’s self-worth is enhanced and the effects of discrimination are reduced when one identifies with a group.^8^ Hendricks et al.^16^ referred to this idea as ‘group-level coping’.

Therefore, TGD individuals experiencing gender incongruence and mental distress often seek gender-affirming healthcare (socially and/or medically), which aims to create greater congruence between sex assigned at birth and self-identified gender.^17^ Furthermore, this forms part of a broader transitioning process which could also involve social, legal, medical and surgical affirmation.^18^ There has been an increased movement towards gender-affirming healthcare as well as towards gender-affirmative training for healthcare professionals.^14^

Although these movements are helpful in addressing the broad range of concerns for TGD individuals and acknowledging the diversity of humans, TGD individuals often perceive the healthcare setting as stigmatising.^19^ When social control is enforced via categorising, stereotyping and the rejection of differences, this is associated with stigma.^20^ According to Hughto et al.,^19^ stigma can operate at three levels, namely an ‘individual level’ (thoughts and behaviour), ‘interpersonal level’ (community interactions) and at an ‘organisational level’ (laws, policies and institutional practices). While homo/transphobic violence is widely described, many TGD individuals feel that discriminatory and inexperienced healthcare providers are a major barrier to accessing care and support.^14^ Therapists surrounded by predominantly cisgender narratives and who are inexperienced in working with TGD individuals are left uncertain of how to best help TGD individuals.^14^

In sub-Saharan Africa, 50% of countries criminalise same-sex relationships.^21^ These countries are conservative and uphold heteronormative ideals.^22^ Same-sex relationships are considered to be un-African.^23^ The South African Constitution is considered as progressive and inclusive. However, TGD individual continues to experience stigma and discrimination. Discrimination against TGD individuals has long been tied to equal accessibility of healthcare services. Furthermore, in the South African context, TGD individuals often experience healthcare workers as discriminatory and hostile because of cisnormative microaggressions.^3^ McLachlan et al.^5^ explain cisnormativity as the assumption that there are only two fixed genders and that gender always reflects the individual’s sex as assigned at birth. Cisnormativity goes further and serves to regulate sexuality as well as gender. In the South African context, McLachlan^5^ makes mention that gender-affirming hormones are included in the National Health Departments’ Essential Medicine List. However, access to these hormones and other healthcare services are only provided by a few hospitals in South Africa because of the unavailability of endocrinologists and the lack of training in gender-affirming healthcare. Statistics indicated that in 2019, over 3 million of the 58 million South Africans presented as gender non-conforming. This is challenging, as in South Arica, the Western binary concept is still prevalent.^24^

Given the above, being empathic is essential when working with TGD individuals. When therapists or clinicians are able to empathise with the negative experiences of TGD individuals, better rapport is established.^6^

Within the TGD community, trust between the individual and healthcare practitioner appears to be one of the key concerns. Individuals who are gender-diverse fear discrimination and uncertainty regarding whether the healthcare setting is accommodating. This results in reluctance to self-disclose and concern over whether they will be receiving quality care.^25^

Limited training both at an undergraduate and postgraduate level restricts the number of trained professionals.^17^ A significant challenge facing our unit at Chris Hani Baragwanath Academic Hospital (CHBAH) is limited staffing. The unit encourages and welcomes new individuals; however, limited resources make this challenging. In light of the above, South African research on TGD interventions is essential. This study highlights the importance of group intervention in particular. To the best of our knowledge, no research was found on existing TGD psychology group interventions in a South African public health context.

Luthando Clinic at CHBAH provides trans and gender-diverse (TGD) individuals with a setting in which they can access gender-affirming healthcare and psychiatric and psychological services. Greater health-seeking behaviours are encouraged through the provision of a non-judgemental space within a healthcare setting, and a relational approach is central to the therapeutic space. The multidisciplinary team (MDT) working with TGD individuals consisted of a psychiatrist, two psychologists and nursing staff. Individuals were referred to social work and occupational therapy when indicated. The gender identity of the MDT includes two males and two females, and all members identify as cisgender.

Individuals were either self-referred or referred by Gender DynamiX, the first registered Africa-based public benefit organisation to focus solely on TGD communities. The MDT met once a week, wherein newly referred individuals were screened and a clinical interview was conducted. Individuals were screened for mental health conditions such as depression, anxiety, suicidality, substance use, social support, psychoeducation and psychosocial stressors. The importance of screening processes is emphasised by a study conducted by Grobler,^26^ which indicated that transgender adults had an incidence of anxiety of 25.9%, substance use 21.0%, eating or psychotic disorders 2.3% and a lifetime prevalence of mood disorder of 21.2%. Individuals who were assessed as potentially able to benefit from the TGD support group were offered the service and included in the group if they consented. Individuals who were not suitable based on acute psychiatric symptoms (for example, psychosis and acute suicidality requiring admission) at the time of screening and interview were treated by other mental healthcare services until they were able to engage in the TGD support group. The TGD support group was an open group, which met every alternate Monday afternoon. The time frame was an hour, and the group ran over a period of 2 years (2017 and 2018) with the current two facilitators. The two facilitating psychologists met weekly for internal supervision and discussion.

The goal of this group was to provide TGD outpatients (OP) with a source of support and encouragement as they progressed through the gender-affirming process. The theoretical orientation was based on the principles of supportive psychotherapy alongside Irvin D. Yalom’s^27^ theoretical understandings of groups. Supportive psychotherapy principles included empathy, reflection, validation and providing a non-judgemental space.^27^

The TGD support group provided an opportunity for trust to develop between the healthcare practitioner and the TGD individual, which aids in their gender-affirming healthcare. Furthermore, this group created the opportunity for members to build their medical knowledge in gender-affirming healthcare as well as to receive general information.^28^

## Aims and objectives

We aim to report on our experiences on facilitating a TGD support group at CHBAH. This paper will be utilised to inform further groups in our institution and other health settings.

## Research methods and design

### Study design and setting

The study assumes an interpretative paradigm, which emphasises the experiences of the facilitators. The approach is reliant upon the subjective perceptions of the facilitators.^29^ Cohen et al.^30^ explain that from an interpretive paradigm, our realities are created subjectively through the meanings developed interactionally and experientially. This paradigm focuses on observer subjectivity.^31^

Subsequently, researchers and the subject under study cannot be separated.^30^ Throughout the research process, findings emerge through conflicting interpretations of the subject being investigated.^30^

A reflexive thematic analysis was also utilised. This is described by Braun et al.^32^ as a specific type of thematic analysis. The researcher’s subjectivity is seen as an analytic resource. Reflexive thematic analysis consists of six phases, namely: (1) familiarising yourself with the data, (2) generating initial codes, (3) searching for themes, (4) reviewing the themes, (5) defining and naming the themes, and (6) producing the report.^33^ These phases are not intended to be followed rigidly or linearly and should be seen as a flexible process.^32^

### Data collection

This qualitative study was conducted at Chris Hani Baragwanath Academic Hospital in Soweto, from February 2022 to November 2023. The authors reviewed their own supervision notes of the group process from 2017 to 2018 and not that of previous facilitators. Consent was obtained during the intake process. For this study, no participants were involved; therefore, no sampling procedure was utilised. No instruments such as tests, surveys, questionnaires or scales were utilised in this research.

### Data analysis

As researchers, we initially immersed ourselves in our supervision notes and read our data in an active way while searching for patterns and generating a list of ideas. We documented theoretical and reflective thoughts while noting possible themes. During this phase, we discussed our own perspectives, preconceived ideas and thoughts as well as our own subjectivity.^34^ Coding was then performed manually by us to indicate potential patterns. The codes that were vague and inconsistent were discarded. Similar ideas were grouped together as potential themes. These themes were inserted into a diagram to make further sense of their connections. Themes were refined by revisiting the original data and diagram to determine their adequacy. Candidate themes were eliminated, as these were thought to be too diverse. Themes were then further refined and named, and the following themes were identified:

- gaps in the facilitators’ knowledge
- role of facilitators
- misgendering because of gender fluidity
- collective self-esteem
- corrective experience
- basic rights: human rights and patient rights.

### Ethical considerations

Ethical considerations were particularly important in the writing of this article, considering the vulnerability of the TGD population. The facilitators adhered to the principle of confidentiality. This study was a retrospective record review with no identifiers and thus no risk to patients and no need for informed consent. However, upon the intake process, individuals consent to the clinical interview, referral to the group and/or individual psychotherapy, and any future research. Ethical clearance to conduct this study was obtained from the hospital (CHBAH), Department of Health (DOH) and from the University of the Witwatersrand Human Research Ethics Committee (Medical) (No. M210155).

## Results

### Gaps in the facilitators’ knowledge

The first theme that emerged was the cautiousness with which we, the facilitators, entered the group. This is on the backdrop of our limited exposure in working with the TGD population. However, our clinical skills laid the foundation in developing this group. As per PsySSA guidelines for working with sexually and gender-diverse people, we acknowledged the limitations of our knowledge to group members and our commitment to enhancing our understanding.^5^ We drew on concepts such as a non-judgemental stance, transparency, empathy and trust to help us establish the process of this group. The group process and members fostered our learning and development. We collaborated in supervision with regard to our subjective experience of the group.

### Role of facilitators

The group aimed to be a containing and supportive space for voluntary members. The group was found to lessen feelings of isolation and loneliness for individuals as they became a support structure to each other. As facilitators, we found that some of the members needed a safe space where they belonged, as many of their narratives alluded to feeling excluded by the wider ‘gay community’. We fostered inclusivity and a space that was destigmatising and accepting.

However, in contrast to our aim, there was an inter-departmental requirement that TGD individuals be seen for individual psychotherapy for a minimum of 6 months prior to additional gender-affirming healthcare. Although the intent was good in terms of protecting patients, this requirement was outdated and disempowering. Furthermore, this escalated TGD individuals’ distress, anxiety and disappointment, as it prolonged their gender-affirming process. This also elicited much discomfort in us, as it goes against current standards of care^2^ that psychologists are not gatekeepers in whether or not the TGD individuals transition to their self-identified gender. Over time, we were able to be transparent in the group about this discomfort and our own processes.

This also led to some group members being ambivalent about the group. For some, the group became an obstacle in their gender-affirming healthcare process. However, they voiced that the group was a safe enough space to explore these thoughts. In addition, other members were able to engage with this and outlined that the group also provided a therapeutic space. Currently, all departments adhere to the updated standards of care^2^ and best practice. Therapy (group and/or individual) is voluntary.

### Misgendering because of gender fluidity

Upon reflection, we realised how mindful we had to be regarding correct pronoun use. However, occasionally, we made errors which became part of the group process and part of our own development. We experienced that some members were open to engage us regarding misgendering, but perhaps the other members were less so because of our professional roles. We reflected as to why some members would be more tolerant towards being misgendered. This is perhaps symbolic of their external experiences whereby they feel they do not have a voice or a sense of control to be more assertive.

In addition, we noticed that group members would misgender each other. However, there was a sense of safety and trust among members to engage each other around this. Upon further reflection, we had to process whether misgendering would unconsciously contribute to stigma.

### Collective self-esteem

Coming from a background of being discriminated, members often presented with decreased self-esteem. As the group progressed, members positively identified with each other, which improved their self-esteem. Positively identifying with one’s social group is referred to as collective self-esteem.^35^ Overall, this created a sense of ease for members and facilitators alike. This allowed us to occasionally assume the role of observers as they experientially created a cohesive space. By means of their narratives, they allowed us to access their experiences of being unsupported, lonely and stigmatised. This leads to an increased sensitivity within us and an awareness of their needs. At times it was challenging to maintain neutrality as we struggled to find a balance between meeting these needs and reinforcing their thoughts of feeling disempowered and hopeless with regard to being accepted.

### Corrective experience

Although this population is subjected to discrimination by healthcare workers, our perception is that the group offered them a corrective experience. This was fostered by our openness to tolerate their frustrations and remain constant. In addition, members offered a corrective experience to each other by being accepting and empathic. This in turn shifted the group to be a more process-orientated group as the members were less guarded. In addition, although we started the process tentatively and subjected some members to misgendering, we experienced the group as a corrective experience for us as well.

### Basic rights: Human rights and patient rights

The extent of the infringement on their basic rights within the healthcare system as well as outside emerged. With regard to human rights, South Africa is progressive. However, TGD individuals continue to experience discrimination and stigmatisation.^36^ This relates to access to healthcare, employment, legal documentation (as in name and gender marker changes), travel and access to bathrooms. The above being out of our control reinforced our sense of helplessness. Their perceived lack of control fed into our own lack of agency with regard to the above. It is important to note that, currently, TGD individuals at CHBAH are no longer required to attend psychotherapy before being referred to the Department of Endocrinology. This shift has resulted in the rights of TGD individuals being acknowledged.

## Discussion

The key findings include gaps in our knowledge, the role of the facilitators, misgendering, collective self-esteem, corrective experience and basic rights. The PsySSA guidelines^2^ encourage professionals to acknowledge and enhance their understanding of TGD individuals. Furthermore, these guidelines clearly inform us that psychologists are not gatekeepers during the gender-affirming process, which helped inform our role as facilitators. Although misgendering has negative effects on TGD individuals,^6^ we experienced the members as open to engaging the facilitators around this theme. The group members’ narratives resonated with each other, which fostered a collective self-esteem^35^ and provided a corrective experience to both facilitators and members. It is imperative that public health aligns itself with the PsySSA guidelines to protect the human rights of TGD individuals.

The group space evolved as members were more open, less guarded and more receptive to receiving support. Furthermore, in relation to feeling isolated and lonely, some members created a support network outside of our group. This is in keeping with Yalom’s^27^ principles of instillation of hope and development of socialising techniques. As the process unfolded, members were perceived to be less despondent, as there was a sense of inclusion and identification with others that decreased their sense of isolation (universality). It emerged in supervision that those members who attended both individual and group psychotherapy displayed improved insight, including improved self-esteem and agency.

## Limitations

Firstly, being an open group, attendance was inconsistent. This inconsistency can be accounted for by members’ financial constraints, as individuals from rural areas have limited facilities and are disadvantaged by logistics. Furthermore, this could also be accounted for by an unconscious resistance, as they had less autonomy with regard to attending group psychotherapy. Our initial idea was to have a more process-oriented group. However, it is challenging to have an open group that is process-based. Furthermore, we had to meet the needs of the members, which were initially around content and discussing practical issues (imparting information). We were mindful that this is an integral part of their gender-affirming healthcare. Secondly, retrospective reflexive studies are subjective in nature by implication. Lastly, the benefits of this group were not objectively measured but emerged in the narrative.

## Recommendations

Upon reflection, we recognise that it is important for facilitators to consider self-disclosing their gender identity. The lack of limited undergraduate and postgraduate training in transgender issues restricts trained healthcare professionals.^24^ It is recommended that healthcare professionals access supervision, be aware of their own biases, own knowledge gaps and be open to allowing TGD individuals to teach us. There is a need for increased involvement of other MDT members in the treatment of TGD individuals. Members requested separate male and female groups. This could be an important future consideration, as well as considering a group for non-binary individuals. It is equally important to have a co-facilitator and receive ongoing supervision. It is recommended to invite group members to co-author future publications and/or to read these publications to offer their valuable input.

## Conclusion

This group fostered our learning and development. We became aware that the role of reparation was central to the group process. High levels of stigma, rejection and discrimination burden the health of TGD individuals. When we reflected on TGD individuals coming from a background of being rejected, abandoned and being invisible, it increased our awareness of the needs of the TGD population and fostered more empathy. We became ‘containing objects’ as the process developed, as evidenced by members returning after initiating hormones. All groups should be on a voluntary basis and all MDT members should be aligned to the guidelines and standards of care.
